# Camera trap and questionnaire dataset on ecosystem services provided by small carnivores in agro-ecosystems in South Africa

**DOI:** 10.1016/j.dib.2018.03.071

**Published:** 2018-03-22

**Authors:** Samual T. Williams, Naudene Maree, Peter Taylor, Steven R. Belmain, Mark Keith, Lourens H. Swanepoel

**Affiliations:** a Department of Zoology, School of Mathematical & Natural Sciences, University of Venda, Private bag X5050, Thohoyandou 0950, South Africa; b Department of Anthropology, Durham University, Durham DH1 3LE, United Kingdom; c South African Research Chair on Biodiversity Value & Change, University of Venda, Private bag X5050, Thohoyandou 0950, South Africa; d School of Life Sciences, University of KwaZulu-Natal, Private Bag X54001, Durban 4000, South Africa; e Natural Resources Institute, University of Greenwich, Chatham Maritime, Kent, United Kingdom; f Eugène Marais Chair of Wildlife Management, Mammal Research Institute, University of Pretoria, 0002, South Africa

## Abstract

This dataset includes data derived from camera trap surveys and questionnaire surveys relating to small carnivores in agro-ecosystems in the Vhembe Biosphere Reserve, South Africa. The data were collected as part of the study “Predation by small mammalian carnivores in rural agro-ecosystems: An undervalued ecosystem service?” (Williams et al., 2017a) [1]. Camera trap locations were stratified by land use: settlement, crops, and grazing areas. The camera trap data provide an insight into the ecology of the nine species of small carnivores that were recorded: striped polecat (*Ictonyx striatus*), honey badger (*Mellivora capensis*), large-spotted genet (*Genetta maculata*), African civet (*Civettictis civetta*), slender mongoose (*Galerella sanguinea*), Meller's mongoose (*Rhynchogale melleri*), Selous' mongoose (*Paracynictis selousi*), white tailed mongoose (*Ichneumia albicauda*), and dwarf mongoose (*Helogale parvula*). We also recorded domesticated animals such as domestic cats (*Felis catus*), domestic dogs (*Canis lupus familiaris*), and cattle (*Bos taurus*) on the camera traps. The questionnaire data are comprised of responses of stakeholders to questions regarding the impacts of these species on rural farming communities. In the accompanying data repository hosted on Figshare (doi 10.6084/m9.figshare.4750807, (Williams et al., 2017b) [2]) we provide raw data, along with processed data and R code used to analyse these data to determine the impact of land use and domestic animals on the species richness and occupancy of small carnivores in rural agro-ecosystems (Williams et al., 2017a) [1].

**Specifications Table**

**Table:** 

Subject area	*Biology*
More specific subject area	*Conservation biology, ecology*
Type of data	*Text file, shapefile, R code*
How data was acquired	*Camera traps, questionnaires*
Data format	*Raw, processed*
Experimental factors	*Camera trap data were stratified by land use*
Experimental features	*Camera trap surveys, questionnaire surveys*
Data source location	*Ka-Ndengeza (S23.310028, E30.409812) and Vyeboom (S23.151735, E30.392782) villages, Limpopo Province, South Africa*
Data accessibility	*Data are available from Figshare (doi:10.6084/m9.figshare.4750807;*https://figshare.com/articles/Small_carnivore_ecosystem_services_data/4750807*)*
Related research article	*This is a companion article to**[1]*

**Value of the data**

• The raw camera trap data could be useful for studying the biodiversity and distribution of small carnivores in agro-ecosystems.
• The processed camera trap data may be useful for the study of small carnivore occupancy.
• The questionnaire data may be of interest to researchers studying the opinions of people towards wildlife.
• These data could be compared with other data collected in protected areas, other geographic locations or among other groups of stakeholders, and could contribute to spatial data sets (e.g. Global Biodiversity Information Facility).
• This could contribute towards scientific research or policy documents, for example the International Union for Conservation of Nature and Natural Resources (IUCN) Red List of Threatened Species, The Red List of Mammals of South Africa, Swaziland and Lesotho, South Africa's National Biodiversity Strategy and Action Plan, or local wildlife management plans.

## 1 Data

**Fig. 1:**
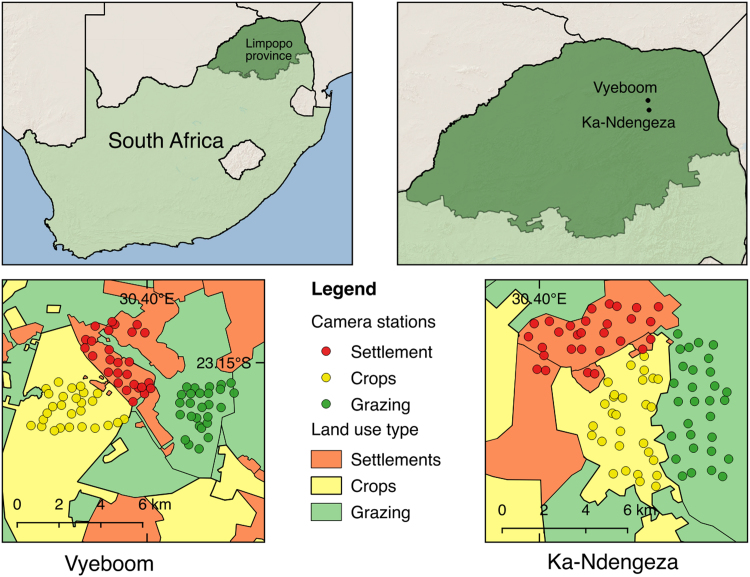
Location of the study sites and the camera traps, showing the settlement, crops and grazing land use types.

**Fig. 2:**
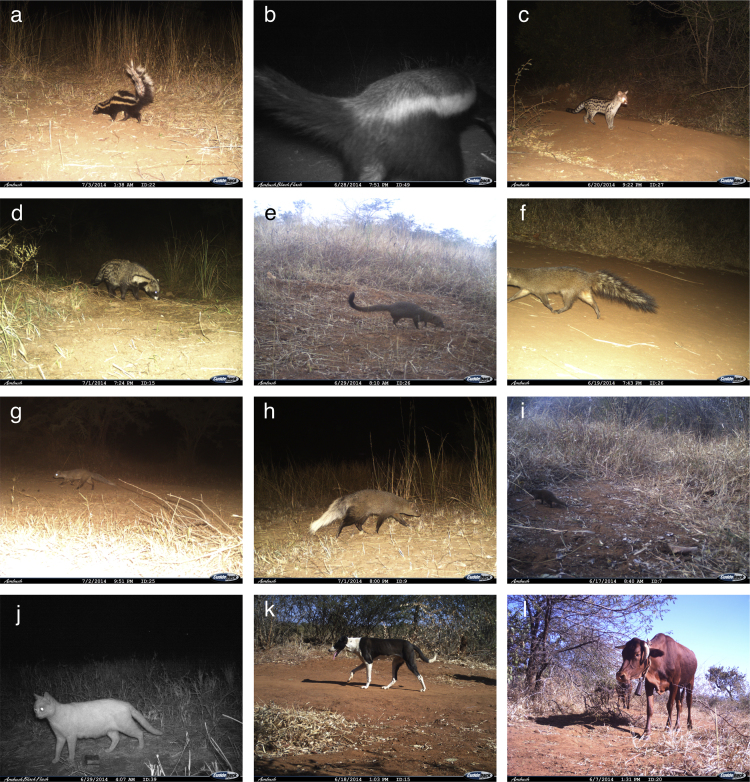
Example photographs collected during the camera trap surveys. These images show a) striped polecat, b) honey badger, c) large-spotted genet, d) African civet, e) slender mongoose, f) Meller's mongoose, g) Selous' mongoose, h) white tailed mongoose, i) dwarf mongoose, j) domestic cat, k) domestic dog, and l) domestic cow.

We provide data collected using camera trap and questionnaire surveys conducted in agro-ecosystems (Fig. 1). Data collection focussed on small carnivores, defined as members of the order Carnivora with a body mass under 15 kg [1]. A total of nine species of wild small carnivores, and two species of domesticated carnivores, were detected (Table 1), along with domestic cattle (Fig. 2). The data were collected in two villages in Limpopo province, South Africa, and camera trap locations were stratified by land use (settlement, crops, and grazing areas).

**Table 1:** Summary of photographs of carnivore species collected during the camera trap study. The table is ordered according to family level (all capitals).

		Number of independent detections per 1000 camera trap days						
		Ka-Ndengeza			Vyeboom			
Common name	Scientific name	Settlement	Crops	Grazing	Settlement	Crops	Grazing	IUCN Red List [10]
CANIDAE								
Domestic dog	*Canis lupus familiaris*	9324	1270	308	5160	201	37	
								
MUSTELIDAE								
Striped polecat	*Ictonyx striatus*	0	0	5	0	8	0	Least concern
Honey badger	*Mellivora capensis*	0	0	0	0	0	6	Least concern
								
FELIDAE								
Domestic cat	*Felis catus*	324	0	10	720	0	6	
								
VIVERRIDAE								
Large-spotted genet	*Genetta maculata*	0	643	217	22	173	228	Least concern
African civet	*Civettictis civetta*	0	0	0	0	8	0	Least concern
								
HERPESTIDAE								
Slender mongoose	*Galerella sanguinea*	0	254	25	0	148	86	Least concern
Meller's mongoose	*Rhynchogale melleri*	0	48	0	0	0	0	Least concern
Selous' mongoose	*Paracynictis selousi*	0	71	0	0	33	0	Least concern
White tailed mongoose	*Ichneumia albicauda*	0	151	0	27	8	19	Least concern
Dwarf mongoose	*Helogale parvula*	0	32	0	4	4	31	Least concern

## 2 Experimental design, materials, and methods

### 2.1 Study area

We conducted the study at two villages in the Vhembe Biosphere Reserve, South Africa: Ka-Ndengeza (S23.310028°E30.409812°), and Vyeboom (S23.151735°E30.392782°) (Fig. 1). Both sites receive an annual rainfall of 700–800 mm per year, with a hot wet season from October to March and a cool dry season from May to August [3]. Natural vegetation is classified as Granite Lowveld and Gravelotte rocky bushveld [4]. Vegetation is characterised by tall shrubs with few trees to moderately dense low woodland on the deep sandy uplands dominated by *Combretum zeyheri* and *Combretum apiculatum*. Low lying areas are characterised by dense thicket to open Savanna with *Senegalia (Acacia) nigrescens, Dichrostachys cinerea*, and *Grewia bicolor* dominating the woody layer, particularly the Granite Lowveld [4].

Three major land-use types were identified in each of the villages. First, the settlement areas were used for residential purposes (hereafter settlements) [5]. The majority of households had large gardens (50–80 m × 40–80 m) which were used to grow crops (maize (*Zea mays*), peanuts, beans (*Phaseolus vulgaris*), ground nuts (*Arachis hypogaea*), avocados mangoes, bananas, litchis, and oranges), and to overnight livestock (cattle, donkeys, sheep, goats, and poultry). The second land-use type identified was cropping areas (hereafter crops). Residents of both villages practiced either rotational cropping (maize, ground nuts, and beans) or intercropping (maize, beans, and pumpkins (*Cucurbita* spp.)). Land preparation was usually by manual labour, and preparation typically began in October or November, while planting commenced in early December. Harvesting of crops occurs in February until late April (crop dependant). Farmers reported yields varying between 5–20 bags (each bag weighing 50 kg) of maize and 3–10 bags of ground nuts (Swanepoel, unpublished data). Crop residues were typically used for livestock fodder. The third land-use type was the grazing areas (hereafter grazing), which comprised of short grass, shrubs and tall trees. In addition to communal grazing of livestock, these grazing areas also served for areas where firewood were collected and informal hunting took place. Due to poor land-management practices, however, the grazing areas were typically overgrazed, with woody plants (*D. cinerea*) replacing shrubs and grass, typically in low-lying areas and drainage lines.

### 2.2 Camera trapping

We divided each study area into a settlement area, cropping area and grazing area, based on recent satellite imagery [6], which was then overlaid with a regular spaced grid with a cell size of 300 × 300 m (9 ha). The size choice of the grid cells was guided by the median home range size of small carnivores expected to inhabit the study areas [1], to adhere to the independent assumptions of occupancy models [7]. We deployed one camera trap in each grid, which resulted in an average spacing between camera traps of 193 m. Camera traps were set to record 24 h per day, with a 30 s delay between detections, continuously for 10–12 days. We deployed camera traps at roads, drainage lines, and well established animal paths. We placed cameras around 30 cm above the ground, and cleared vegetation in front of camera traps to reduce the number of false triggers.

In the settlement grid cells we deployed 27–30 infra-red flash cameras (Cuddeback Ambush 1194), as these were less disruptive to the inhabitants of villages than cameras using a visible light flash, while in the crops and grazing areas we deployed 55–60 xenon flash cameras (Cuddeback Ambush 1170). Camera traps were deployed between 2–26 June 2014 at Ka-Ndengeza and 17 June–27 July 2014 at Vyeboom. This resulted in a camera trapping effort of 810 trap days in Ka-Ndengeza and 738 trap days in Vyeboom. To classify land use we first digitized the different land-use types using satellite imagery from Google Maps [6], which we later ground-truthed. We classified crops as either active fields, i.e. still showing agricultural activity, or as abandoned fields. For each camera trap we calculated the percentage of crops, grazing and settlement that comprised the camera trapping grid cell in which each camera trap was located. Camera trap images were catalogued using Camera Base version 1.7 [8].

### 2.3 Questionnaires

We assessed the opinions of community members towards small carnivores using a structured questionnaire (based on the questionnaire used by Holmern and Røskaft [9]), completed by a total of 127 respondents (*n* = 58 in Ka-Ndengeza and *n* = 69 in Vyeboom). For each camera trap the inhabitants of the nearest household were sampled, but when this was not possible another nearby house was selected. If several households were equidistant to a camera trap, we sampled one of these households at random. Photographs of small carnivore species were provided to ensure that the species were correctly identified. We asked interviewees whether they had seen each species of carnivore, if they were good for the community, if they kill rodents, if they had impacted the respondents negatively, and if they were aware if any small carnivore species that are killed by people. The reasons for any positive and negative impacts of the species were also recorded. We also asked whether interviewees consider poultry to be an important source of protein.

### 2.4 Data availability

Raw camera trap and questionnaire data are available in the online repository [2]. We processed these data to allow us to model the influence of land use on carnivore species richness, and model the effect of the relative abundance index of domestic animals on carnivore occupancy (see [1] for details of data processing and analysis). The processed data and R code are also available in [2].

### 2.5 Ethical approval

Ethical approval for the study was provided by the Ethics Committee of the University of Venda (approval number SMNS/14/ZOO/03/2803). We also obtained consent to interview community members of Ka-Ndengeza and Vyeboom from each community Chief in addition to community members. We informed each respondent that anonymity would be maintained, and obtained written consent from interviewees.

## Appendix A Supplementary materials

The following KMZ files contain the Google maps of the most important areas described in this article.

Map KMZ file containing the Google map.
