# Supplementary material for: Camera trap and questionnaire dataset on ecosystem services provided by small carnivores in agro-ecosystems in South Africa
Source: Data Brief. 2018 Mar 22;18:753–9. doi: 10.1016/j.dib.2018.03.071 (PMC5996287; doi:10.1016/j.dib.2018.03.071)
Supplement: Supplementary file 1 — Transparency document [file mmc1.zip › Declarations of interest.pdf]

Declarations of interest: None
